# Supplementary material for: Effects of Diets Enriched with Conventional or High-Oleic Canola Oils on Vascular Endothelial Function: A Sub-Study of the Canola Oil Multi-Centre Intervention Trial 2 (COMIT-2), a Randomized Crossover Controlled Feeding Study
Source: Nutrients. 2022 Aug 18;14(16):3404. doi: 10.3390/nu14163404 (PMC9416081; doi:10.3390/nu14163404)
Supplement: Supplementary file 1 [file nutrients-14-03404-s001.zip › nutrients-1844385-supplementary.pdf]

Table S1. Comparison of baseline characteristics in subsample and full sample.

	Mean: subsample	Mean: Total sample	T(df)	95% CI	*p*
Age (years)	42.6	45.2	0.97 (46.80)	−2.83 to 8.07	0.34
BMI (kg/m^2)	32.43	31.90	−0.47 (45.26)	−2.78 to 1.72	0.64
Waist (cm)	106.2	103.9	−0.93 (50.38)	−7.17 to 2.62	0.36
TG # (mmol/L)	1.39	1.42	0.20 (44.05)	0.35 to 0.33	0.84
HDL-C # (mmol/L)	1.21	1.34	1.58 (37.69)	−0.03 to 0.23	0.12
SBP (mmHg)	123.9	118.6	−1.95 (50.07)	−10.75 to 0.16	0.06
DBP (mmHg)	85.2	76.7	−4.32 (54.26)	−12.32 to −4.50	<0.001 *
Glucose # (mmol/L)	5.31	5.33	0.15 (58.10)	−0.04 to 0.04	0.88
Insulin # (pmol/L)	82.39	86.50	0.42 (48.57)	−0.18 to 0.28	0.67
HOMA_IR #	2.80	2.95	0.43 (51.82)	−0.19 to 0.29	0.67

non-normally distributed variables are presented as geometric means; Welch's independent samples t-tests were used to test for significant differences; BMI, body mass index; TG, triglycerides; SBP, systolic blood pressure; DBP, diastolic blood pressure; HDL-C high-density lipoprotein cholesterol; HOMA-IR, homeostatic assessment of insulin resistance; CI, confidence interval.
